# Outcomes of Intercalary Endoprostheses as a Treatment for Metastases in the Femoral and Humeral Diaphysis

**DOI:** 10.3390/curroncol29050284

**Published:** 2022-05-13

**Authors:** Michal Mahdal, Lukáš Pazourek, Vasileios Apostolopoulos, Dagmar Adámková Krákorová, Iva Staniczková Zambo, Tomáš Tomáš

**Affiliations:** 1 First Department of Orthopedic Surgery, St. Anne’s University Hospital, 65691 Brno, Czech Republic; michal.mahdal@fnusa.cz (M.M.); lukas.pazourek@fnusa.cz (L.P.); vasileios.apostolopoulos@fnusa.cz (V.A.); 2 Faculty of Medicine, Masaryk University, 60177 Brno, Czech Republic; iva.zambo@fnusa.cz; 3 Clinic of Comprehensive Cancer Care, Masaryk Memorial Cancer Institute, 60200 Brno, Czech Republic; dadamkova@mou.cz; 4 First Pathology Department, St. Anne’s University Hospital, 65691 Brno, Czech Republic

**Keywords:** bone neoplasm, metastasis, diaphysis of long bones, intercalary endoprosthesis

## Abstract

Background: The purpose of this study was to evaluate the implant survival, functional score and complications of intercalary endoprostheses implanted for metastatic involvement of the femoral and humeral diaphysis. Methods: The selected group covered patients with bone metastasis who were surgically treated with an intercalary endoprosthesis between 2012 and 2021. The functional outcome was evaluated with the Musculoskeletal Tumor Society (MSTS) scoring system, and complications were evaluated by using the failure classification for prosthetics designed by Henderson. Results: The mean follow-up was 29.8 months. In our group of 25 patients with 27 intercalary endoprostheses (18 femurs, 9 humeri), there were 7 implant-related complications (25.9%), which were more common on the humerus (4 cases, 44.4%) than on the femur (3 cases, 16.7%). Only type II failure—aseptic loosening (5 cases, 18.5%)—and type III failure—structural failure (2 cases, 7.4%)—occurred. There was a significantly higher risk of aseptic loosening of the endoprosthesis in the humerus compared with that in the femur (odds ratio 13.79, 95% confidence interval 1.22–151.05, *p* = 0.0297). The overall cumulative implant survival was 92% 1 year after surgery and 72% 5 years after surgery. The average MSTS score was 82%. The MSTS score was significantly lower (*p* = 0.008) in the humerus (75.9%) than in the femur (84.8%). Conclusions: The resection of bone metastases and replacement with intercalary endoprosthesis has excellent immediate functional results with an acceptable level of complications in prognostically favourable patients.

## 1. Introduction

The increasing incidence of cancer, alongside improvements in the treatment of advanced stages of the disease, has led to an increase in the number of patients with advanced cancer and the significant prolongation of their survival. The skeleton is the third most common site for metastases, a fact that significantly affects a patient’s quality of life. Therefore, the management of bone metastases and so-called skeletal-related events has increased in importance [1].

We believe the basic principle of bone metastasis surgery is to ensure that the chosen reconstruction ‘outlives’ the patient and not vice versa [2,3]. Therefore, the basic criterion for indicating the optimal surgical solution is the patient’s prognosis [1,4,5]. Estimating a prognosis may be difficult, and interdisciplinary cooperation is often necessary [1,6]. There is dependence on the type of primary tumour, the presence of only bone or visceral metastases, the options of systemic oncological treatment, the overall condition and comorbidities [1,4,5]. Usually, the goal of our treatment is palliative; in rarer cases of solitary metastases and oligometastatic involvement in some types of tumours, curative treatment may also be considered.

In the case of diaphyseal metastatic involvement in patients with a poor prognosis and an expected survival between 3 and 6 months, a sufficient solution is to stabilise the impending or already-established pathological fracture with an intramedullary nail [1,3,4]. In patients with a survival prognosis of 6–12 months, an intralesional procedure with osteosynthesis and cementoplasty is indicated to achieve the most durable construction [3,7]. In patients with a survival prognosis of >1 year, resection of a metastatic lesion and replacement with an endoprosthesis is indicated [1,8,9,10]. In the case of a simple stabilisation of an impending or established pathological fracture, or after a non-radical operation, we could reduce the risk of local progression by providing supplementary postoperative radiotherapy [11]. Resection and replacement with an intercalary endoprosthesis seems to be an ideal solution for prognostically favourable patients with metastatic impairment in the diaphysis of long bones [2,7,12,13,14].

This procedure has several advantages. Tumour resection prevents the local progression of metastasis treated by osteosynthesis alone and reduces the risk of recurrence compared with intralesional procedures [7,15]. The technique of implanting the intercalary endoprosthesis itself is relatively easy and fast. Reconstruction with an intercalary endoprosthesis is immediately strong enough to allow for early mobilisation, full-impact activities and the rapid recovery of limb function [12,14]. Mechanical strength is higher than simple stabilisation by an intramedullary nail [16]. The risk of failure after 1 year, especially in the lower limb, is lower than with a construction that combines bone cement with osteosynthesis [12]. There are no complications associated with the problematic healing of biological reconstructions, which usually require long-term limb relief [12,17,18]. Like other techniques, an intercalary endoprosthesis has several limitations and complications. The main limitation is the option of anchoring stems of the appropriate length, proximally and distally to the level of the resection. The main complications then include aseptic loosening and structural failure of the implant [12,13,19,20,21].

The scope of this study is to evaluate the implant survival, functional scores and complications of intercalary endoprostheses implanted in our database for metastatic involvement of the femoral and humeral shaft.

## 2. Materials and Methods

In this retrospective study, we analysed the records of patients with metastatic skeletal involvement in our musculoskeletal oncology centre from 2012 to 2021. We excluded a cohort of patients with metastatic long bone disease who had been referred to our department for treatment. The inclusion criteria were patients with metastases to the diaphysis of the long bones, sparing of the joint above and below, who were surgically treated with a segmental intercalary endoprosthesis (from Beznoska s.r.o. Kladno, Czech Republic; Prospon spol. s.r.o., Kladno, Czech Republic; or Implantcast GmbH, Buxtehude, Germany). The exclusion criteria were expected survival of <1 year and poor general condition. We created a clinical dataset of 25 patients (27 implants) with a diaphyseal defect after the resection of tumours of the femur and humerus. The recorded data included the age, gender, indications for surgery, size, location and histopathology of the tumour, date of surgery, follow-up, complications and functional outcome. Endoprosthetic reconstruction was performed 27 times in 25 patients, comprising 18 (66.7%) femurs and 9 (33.3%) humeri. Two intercalary endoprostheses were used secondarily as a solution to the failure of a previous diaphyseal implant. Our dataset includes 15 men and 10 women with a mean age of 64.4 years (range 49–79 years). The histologic diagnosis, surgical details, radiotherapy, follow-up, complications and Musculoskeletal Tumor Society (MSTS) score for each patient are presented in Table 1.

Tumours were treated using standard oncologic principles. Resection was indicated in patients with solitary metastasis or oligometastasis with a prolonged survival prognosis. All patients had a wide resection of the diaphyseal tumour, as confirmed by postoperative histological evaluation of the resected specimens. Postoperative radiotherapy was indicated in six cases. No patient took drugs that could potentially affect implant fixation.

**Figure 1:**
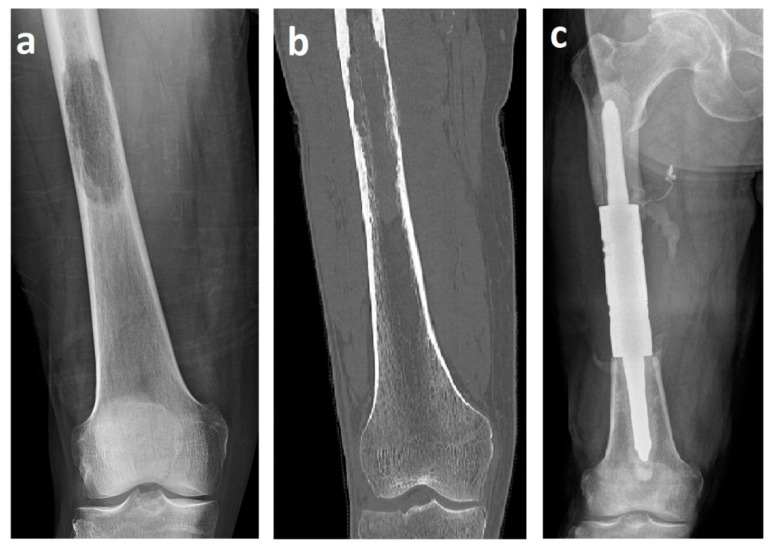
X-ray (**a**) and computed tomography (**b**) images of renal cell carcinoma solitary metastasis to the femoral diaphysis. X-ray image (**c**) after resection with a cemented intercalary endoprosthesis. These images are from patient 2 in Table 1.

The mean length of the resection and bone defect was 11.2 cm (range 8–16 cm). An intercalary endoprosthesis with cemented stems was used in all cases. The implant consists of a central spacer clamped onto a proximal and distal intramedullary stem. Canals were reamed proximally and distally with flexible reamers. Endoprosthetic body segments were placed, and proper rotation was determined by pre-resection marks placed in the proximal and distal aspects of the affected bone. The intramedullary stems were fixed at the proximal and distal bone stumps with polymethylmethacrylate (PMMA). Then, the spacer was assembled in situ with the stems using interconnection screws (Figure 1).

Postoperative complications were recorded, and an oncological follow-up was performed. A routine follow-up evaluation was performed every 3 months for the first 2 years, every 6 months for the next 3 years and then annually. Each follow-up evaluation included clinical examination and imaging methods.

Complications were evaluated by using a classification according to five failure modes for prosthetics proposed by Henderson et al. [22,23]: soft tissue failure (type I), aseptic loosening (type II), structural failure (type III), infection (type IV) and tumour progression (type V). Potential failure was identified by using clinical examination and imaging methods. Function was evaluated by using the MSTS scoring system for the upper and lower extremities [24]. This system includes numerical values from 0 to 5 points assigned for each of the following six categories: pain, function, emotional acceptance, hand positioning, dexterity and lifting ability. The score obtained at the last patient check-up was used for evaluation. The values were added, and the functional score is presented as a percentage of the maximum score possible.

Patient data analysis pertained to implant survival, complications and functional outcomes. Statistical outcomes were measured by using Fisher’s exact test and the Mann–Whitney U test, with the level of significance set at *p* < 0.05. The Kaplan–Meier estimator was used to evaluate the survival of the endoprostheses. We used Minitab data analysis software to evaluate our results.

## 3. Results

The mean follow-up was 29.8 months (range 3–105 months). At the latest examination, 11 (44%) patients had passed away of the disease (DOD), 6 (24%) are continuously disease-free and 8 (32%) are currently alive with the disease. There was no local recurrence of resected bone metastasis recorded during the study period. In 25 patients (92.6%), surgery was indicated as an initial treatment of a tumour. Two patients (7.4%, patients 8b and 13b) were operated on for failed previous individual intercalary endoprostheses. 

**Figure 2:**
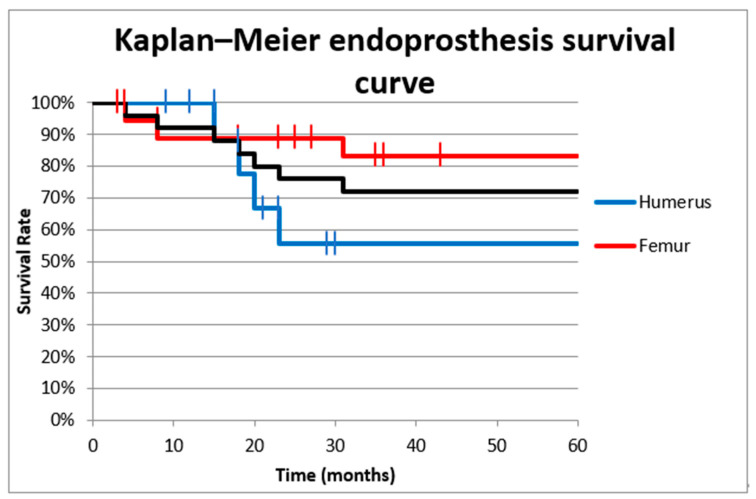
Cumulative survival curve of intercalary endoprostheses for metastases in the femoral and humeral diaphysis. The overall survival 1 year after surgery was 92% (88.8% in the femur and 100% in the humerus). The 5-year overall survival rate was 72% (83.3% in the femur and 55.5% in the humerus).

The survival rate of the intercalary endoprosthesis was evaluated by using the Kaplan–Meier cumulative survival curve. The cumulative overall survival of the diaphyseal implant 1 year after surgery was 92% (88.8% for the femur and 100% for the humerus 100%). The 5-year overall implant survival was 72% (83.3% for the femur and 55.5% for the humerus) (Figure 2). We used univariate analysis to evaluate the 5-year overall endoprosthesis survival based on location (femur or humerus); there was not a significant effect (odds ratio [OR] 4, 95% confidence interval [CI] 0.656–24.36, *p* = 0.175).

Our dataset comprises 25 patients with 27 intercalary endoprostheses (18 femoral, 9 humeral). Seven implant-related complications (25.9%) were recorded—more frequent on the humerus (4 cases, 44.4%) than on the femur (3 cases, 16.7%). Of these seven cases, five (18.5%, patients 7, 14, 17, 21 and 25) had type II failure (aseptic loosening) and two (7.4%, patients 8a and 13a) had type III (structural) failures. Soft tissue failure, infection or tumour progression did not occur. The mean time from surgery to the development of a complication was 17 months (range 4–31 months).

**Figure 3:**
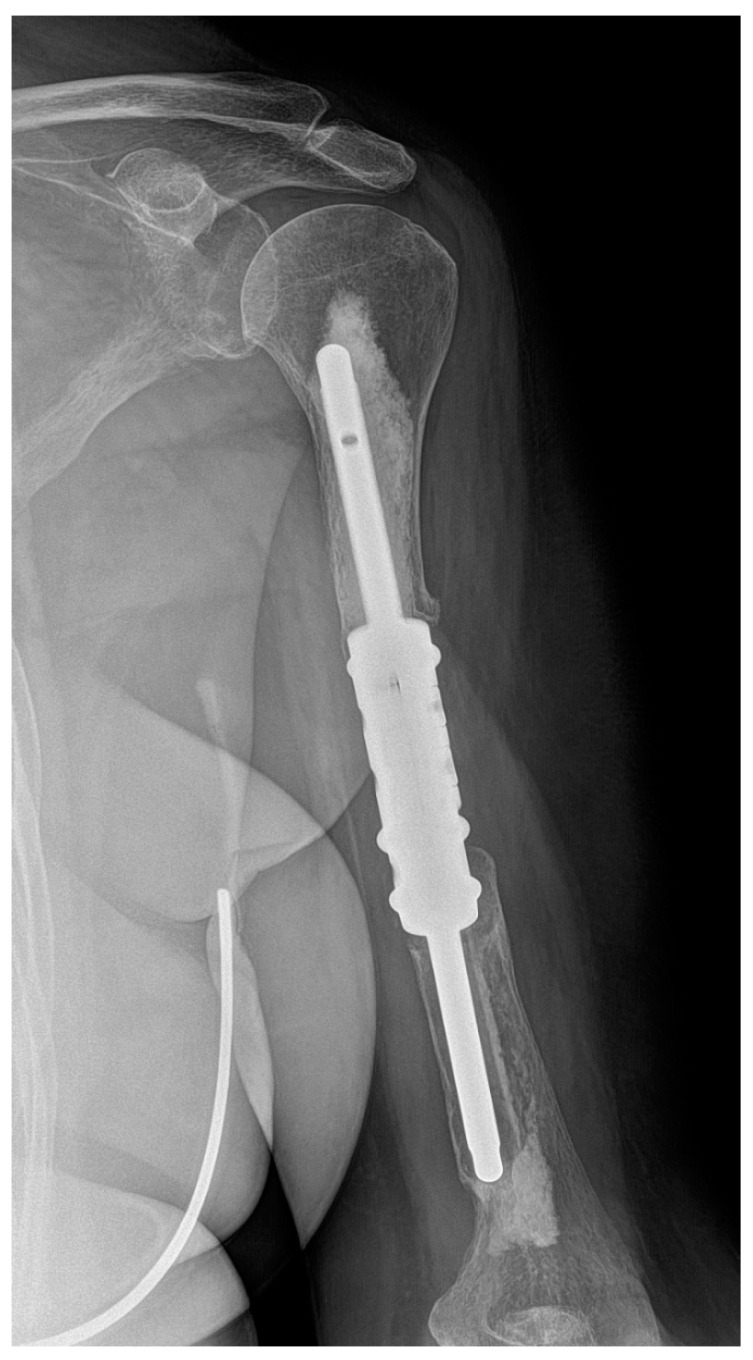
X-ray image of aseptic loosening of the distal stem of the humeral intercalary endoprosthesis. Resection of the metastasis revealed it was a uterine sarcoma solitary metastatic lesion of the left humerus. After 23 months, there was aseptic loosening. Due to the patient’s poor general condition (pulmonary and multiple skeletal metastases) and acceptable functional result (a Musculoskeletal Tumor Society score of 21), revision was not indicated. This image is from patient 7 in Table 1.

Four of the five cases of type II failure were localised in the humerus (patients 7, 17, 21 and 25) and one in the femur (patient 14). Aseptic loosening occurred on average 21.4 months (range 15–31 months) after surgery and was the most common complication in our study (18.5%). In the group of segmental humeral endoprostheses, there were four cases of aseptic loosening (44.4%): the distal stem in three cases and a single proximal stem in one case (Figure 3). There was only one case (5.6%) of aseptic loosening of the proximal stem of the individual femoral intercalary endoprosthesis. However, in cases of type II failure, no reoperation was indicated due to the general condition of the patient and functional status of the limb. There was a significantly higher risk of aseptic loosening of the diaphyseal implant in the humerus compared with the femur (OR 13.79, 95% CI 1.22–151.05, *p* = 0.0297).

**Figure 4:**
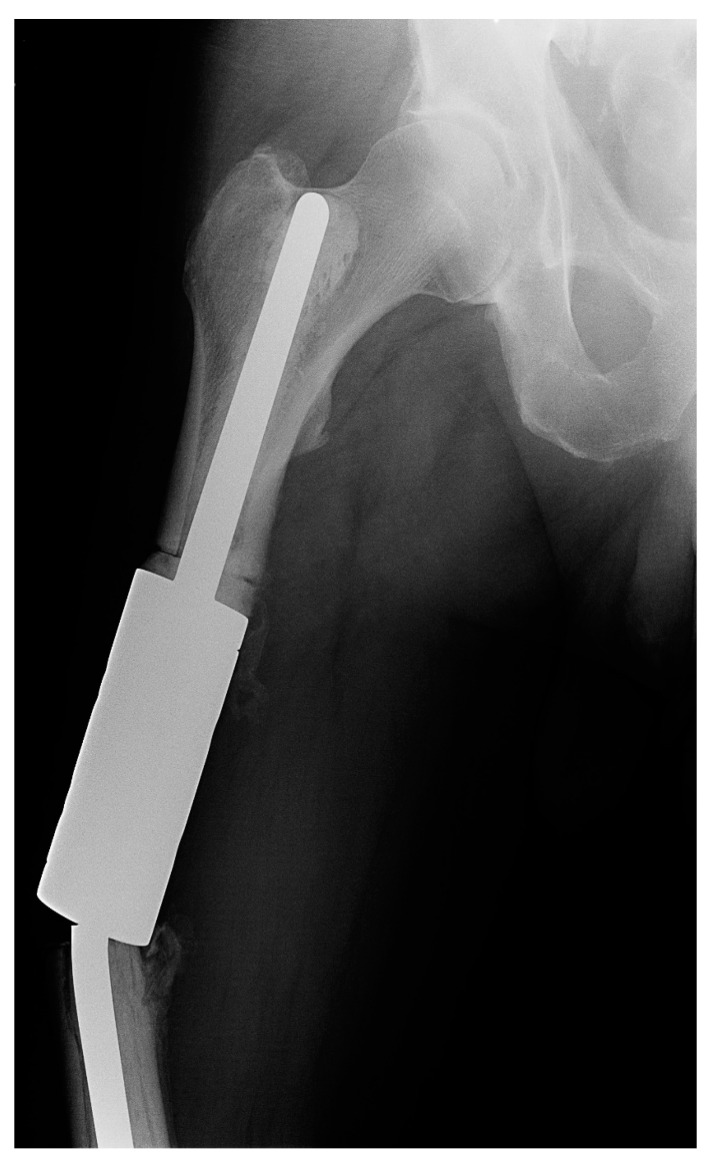
X-ray image of a fracture at the clamp-rod interface (type III failure) of an individual femoral diaphyseal implant for renal cell carcinoma oligometastatic disease. Revision intercalary endoprosthesis was performed. This image is from patient 8 in Table 1.

Both cases of type III failure occurred on the femur (patients 8a and 13a). Both had a fracture of the individual implant (IIIa) that occurred at the clamp-rod interface (overall, 7.4% and 11.1% of femoral reconstructions) (Figure 4). The time to failure was 4 and 8 months from the surgery and required revision with a new spacer clamp.

The complication rate was not significantly associated with the age and sex of the patients, resection size, type of implant, histopathology of the primary tumour, adjuvant radiotherapy or revision procedure.

**Figure 5:**
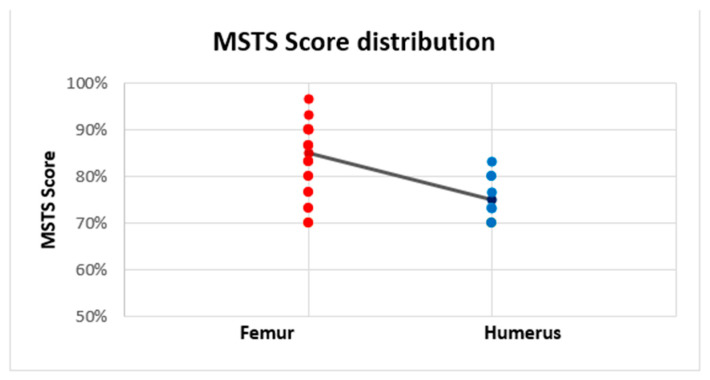
Linear trendline of the Musculoskeletal Tumor Society (MSTS) scores of intercalary endoprostheses for metastases in the diaphysis of femur and humerus. The MSTS score was higher in the femur (84.8%) than in the humerus (75.9%).

The mean MSTS score (evaluated at the last examination) was 82% (range 70–96.7%); the absolute MSTS scores are reported in Table 1. The average MSTS score was 75.9% in the humerus and 84.8% in the femur. The MSTS score was significantly lower in the humerus (median = 1.135, *n* = 9) compared with the femur (median = 1.90, *n* = 18) (Mann–Witney U test, U = 29, z = 2.64, *p* = 0.008), with a medium effect size (r = 0.50) (Figure 5).

## 4. Discussion

Resection and replacement with an intercalary endoprosthesis seems to be an ideal solution for prognostically favourable patients with metastatic impairment in the diaphyseal area indicated for surgery [2,7,12,13,14]. However, even with this reconstruction, researchers have reported a high incidence of complications, especially structural failure and aseptic loosening [19,20]. A significant advantage of a cemented intercalary endoprosthesis in patients with metastatic impairment, and thus mostly limited survival, is immediate stability, the preservation of adjacent joint function, the possibility of early weight-bearing and rapid rehabilitation [12,14].

The overall survival of the diaphyseal implant 1 year after surgery was 92%. The 5-year overall survival was 72%, with significantly better results in the femur (83%) than in the humerus (55%). Aldlyami et al. [25] reported a cumulative overall survival of intercalary endoprosthetic reconstruction of 63% after 10 years. The relatively higher failure rate of intercalary endoprostheses in long-term results is the reason why biological reconstruction is still preferred in cases of primary tumours and younger patients with a good prognosis in the diaphyseal location [12,17,18]. While biological reconstructions achieve a stable construct after the first years of surgical interference, intercalary endoprostheses continue to fail [12,19,26].

According to the Henderson score [22,23], only type II failure (aseptic loosening, 5 cases (18.5%)) and type III failure (structural failure, 2 cases (7.4%)) occurred in our study. There was no soft tissue failure, infection or tumour progression. Compared with other endoprostheses, intercalary endoprostheses have a significantly lower risk of infectious complications. Ruggieri et al. [19] reported no infections in a cohort of 24 patients. Benvenia et al. [20] had only 1 infectious complication in a group of 44 intercalary endoprostheses (2%). Similarly, Büyükdogan et al. [13] reported only 1 infection (4.5%) in their cohort of 22 intercalary endoprostheses.

Aseptic loosening (type II failure) is the most common complication of intercalary endoprostheses [12,13,19,20,21]. It is also the most common complication in our study (18.5%). However, our type II failure rate is significantly lower than in other published studies that are larger and with longer follow-ups, namely, 25% [6], 28.6% [21] and 38% [7]. We found a significantly higher risk of aseptic loosening of the intercalary endoprosthesis in the humerus compared with the femur (*p* = 0.0297). On the contrary, some authors have described the femur as the riskiest area for intercalary endoprosthesis failure (aseptic loosening) [19,20,21]. Ruggieri et al. [19] mentioned that location in the femur is one of the two main risk factors for failure and recommended that this reconstruction be at least reconsidered or even not recommended. However, in line with our experience, many other authors consider the use of intercalary femoral endoprostheses to be a reliable technique with good results [3,5,16,17,20]. Like Ahlmann et al. [14] and Zhao et al. [27], we consider the increased rotational stress in the upper limb area to be the cause of the higher rate of aseptic loosening of humeral stems. The issue of humeral intercalary spacers has been addressed in the literature, mainly by Zhao et al. [7,27]. They have recommended a combination with a bridging plate to eliminate torsional and tensile forces leading to aseptic loosening of the endoprosthesis stems [27] and have even developed an implant connecting the plate to the humeral intercalary spacer [28].

Structural failure (type III) includes implant fracture (type IIIa) and periprosthetic fracture (type IIIb) [22,23]. In our study, there were two implant fractures of femoral diaphyseal endoprosthesis (overall, 7.4% and 11.1% of femoral reconstructions). Concerning load, type IIIa failure is associated almost exclusively with location on the lower limb, especially in the femur. Benvenia et al. [20] reported six cases (14%) with this type of failure at the clamp-rod interface associated with cemented fixation.

**Figure 6:**
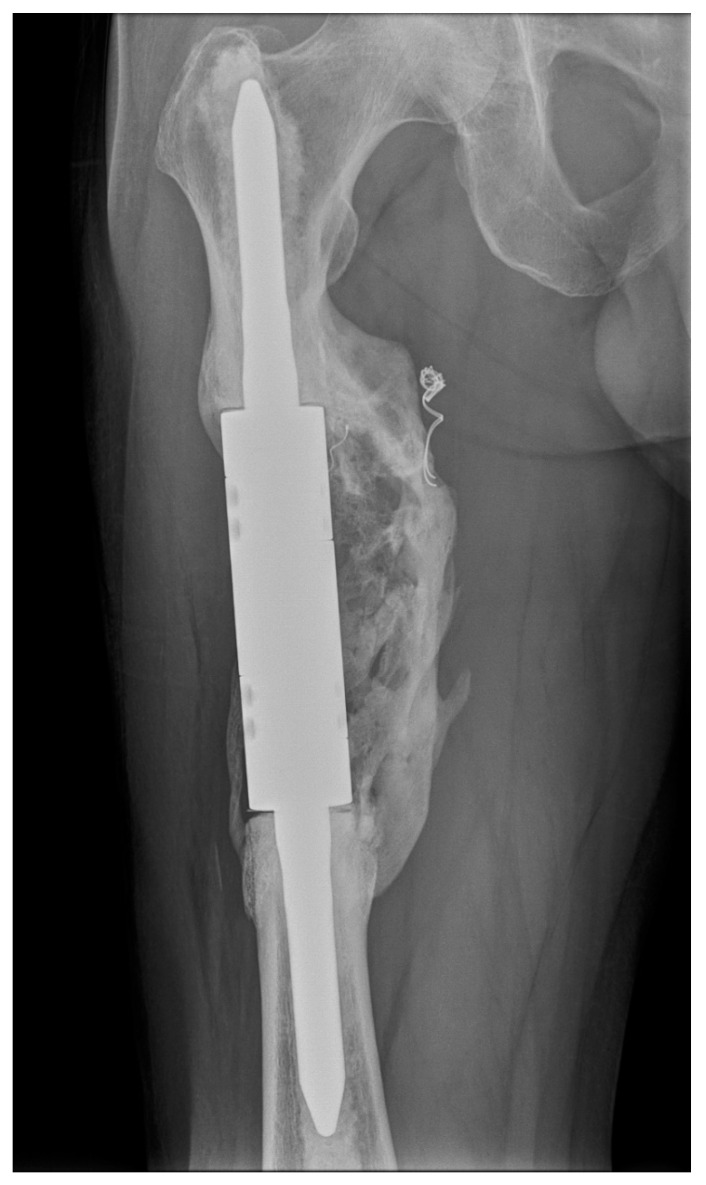
X-ray image of an intercalary endoprosthesis with the formation of heterotopic ossification around the implant after the resection of a renal cell carcinoma metastasis, with a bone bridge connecting the proximal and distal bone fragments. This image is from patient 1 in Table 1.

Other factors mentioned in the literature that may affect the failure of intercalary spacers are the resection length, stem lengths, stem anchoring location, type of fixation (cement versus non-cement), stem adjustment and type of prosthesis (individual versus modular). Ruggieri et al. [19] mentioned a resection length > 10 cm as the second main risk factor for failure. In our cohort of patients, there was not a significant relationship between the risk of failure and the resection length (the average resection length was 11.3 cm (range 8–16 cm)). According to Fuchs et al. [29], there is a contraindication to use intercalary spacers with standard stems to anchor lengths < 5 cm. According to Streitbürger et al. [21], the riskiest shaft anchorage is in the metaphyseal and metadiaphyseal locations. Only cemented stems were used in our group of patients. Cemented fixation is associated with increased postoperative function (MSTS score) and fewer complications [20]. An interesting factor that could minimise the risk of aseptic loosening and thus positively affect implant survival is the formation of heterotopic ossification around the implant, which often forms a bone bridge connecting the proximal and distal bone fragments [14,27,30,31]. We have repeatedly observed this condition forming around the reconstructions after resections of kidney cancer metastases (Figure 6).

We used the MSTS score [24] to evaluate the functional outcome of our patients. The average MSTS score (evaluated at the last follow-up, a mean of 29 months) was 82%, and we found a significantly lower (*p* = 0.008) MSTS score in the humerus (average MSTS score 75.9%) than in the femur (average MSTS score 84.8%). The reason for this difference is aseptic loosening of the stem of the humeral intercalary endoprosthesis, which occurred in four cases (44.4%) with an average interval from implantation of 19 months and was not repaired due to concerns related to the patient’s condition and limb function. The MSTS score expressing the percentage retention of function compared with a fully functional limb was slightly lower in our group compared with other published studies. Ahlmann et al. [14] reported an average MSTS score of 90%, with an average follow-up of 21.6 months. Ruggieri et al. [19] reported a mean MSTS score of 90% for the upper extremity and 86% for the lower extremity, with an average follow-up of 29 months. Büyükdogan et al. [13] published a median MSTS score of 86.9% at a median follow-up of 17 months for their entire cohort. On the other hand, in their multi-centre study of modular intercalary endoprostheses with a mean follow-up of 14 months, Benvenia et al. [20] reported an overall mean MSTS score of 77%. In contrast to our results, some authors have reported poorer functional results in the lower limb area [13,19].

This study has several limitations. First stands the design of the study. This is a retrospective study with a small cohort of patients and a mid-term follow-up. Second, due to the limited dataset, our study lacks multivariate analyses. Third, concomitant systemic therapy was not taken into account, as it does not affect the implant outcome. Considering the limitations, we suggest that adequate results were obtained.

## 5. Conclusions

The results of this study indicate that resection and reconstruction by intercalary endoprosthesis is the method of choice in prognostically favourable patients with metastatic diaphyseal involvement. We consider this technique to be simple and effective, with excellent immediate functional results and an acceptable level of complications. Patients treated with intercalary endoprostheses in the humerus experienced more frequent complications than those treated for lesions in the femur, especially in terms of aseptic loosening. The clinical and functional outcomes in the femur appear to be excellent, with a low rate of complications. 

## Figures and Tables

**Table 1:** Clinical dataset: patient details, complications, functional outcomes, tumor and endoprostheses characteristics.

Patient Number	Location	Diagnosis	Age (Years)	Resection Length (cm)	RT	Prosthesis Type	Follow-Up (Months)	Failure Type	Time to Failure (Months)	Complications	MSTS
1	Femur	Renal cell carcinoma	52	12	Yes	Modular	35			None	28
2	Femur	Renal cell carcinoma	71	15	No	Modular	27			None	29
3	Femur	Renal cell carcinoma	73	10	No	Modular	25			None	27
4	Humerus	Lung cancer	65	9	Yes	Modular	9			None	23
5	Humerus	Renal cell carcinoma	56	9	No	Modular	12			None	25
6	Humerus	Breast cancer	75	8	No	Modular	9			None	24
7	Humerus	Uterine sarcoma	49	10	No	Modular	30	II	23	Aseptic loosening	21
8a	Femur	Renal cell carcinoma	64	11	No	Individual	8	III	8	Spacer failure	21
8b	Femur	Failed spacer	65	11	No	Individual	94			None	27
9	Femur	Renal cell carcinoma	63	8	No	Individual	3			None	22
10	Femur	Breast cancer	79	15	No	Individual	18			None	26
11	Femur	Melanoma	74	12	No	Individual	105			None	27
12	Femur	Renal cell carcinoma	67	10	No	Individual	3			None	23
13a	Femur	Renal cell carcinoma	68	15	No	Individual	4	III	4	Spacer failure	21
13b	Femur	Failed spacer	68	15	No	Individual	81			None	27
14	Femur	Renal cell carcinoma	75	10	Yes	Individual	36	II	31	Aseptic loosening	25
15	Femur	Colon cancer	54	14	No	Modular	8			None	24
16	Femur	Renal cell carcinoma	78	9	No	Modular	23			None	26
17	Humerus	Breast cancer	57	8	No	Modular	29	II	20	Aseptic loosening	22
18	Femur	Renal cell carcinoma	59	16	Yes	Modular	43			None	27
19	Femur	Renal cell carcinoma	61	15	No	Modular	36			None	26
20	Humerus	Renal cell carcinoma	62	9	No	Modular	18			None	23
21	Humerus	Melanoma	53	10	No	Individual	21	II	15	Aseptic loosening	22
22	Femur	Renal cell carcinoma	59	12	No	Modular	65			None	27
23	Femur	Lung cancer	66	11	Yes	Modular	23			None	25
24	Humerus	Lung cancer	71	9	No	Modular	15			None	25
25	Humerus	Breast cancer	55	10	Yes	Modular	24	II	18	Aseptic loosening	21

Abbreviations: MSTS, Musculoskeletal Tumor Society; RT, radiotherapy.

## Data Availability

The data presented in this study are available on request from the corresponding author. The data are not publicly available for privacy reasons.
